# Investigation of the effect of anthropogenic land use on the Pănăzii Lake (Romania) catchment area using Cs-137 and Pb-210 radionuclides

**DOI:** 10.1371/journal.pone.0251603

**Published:** 2021-06-17

**Authors:** Robert-Csaba Begy, Codrin F. Savin, Szabolcs Kelemen, Daniel Veres, Octavian-Liviu Muntean, Cristian V. Malos, Tibor Kovacs

**Affiliations:** 1 Faculty of Environmental Science and Engineering, University of Babes-Bolyai, Cluj-Napoca, Romania; 2 Interdisciplinary Research Institute on Bio-Nano-Sciences, University of Babes-Bolyai, Cluj-Napoca, Romania; 3 Institute of Speology “Emil Racoviță”, Romanian Academy, Cluj-Napoca, Romania; 4 Institute of Radiochemistry and Radioecology, University of Pannonia, Veszprém, Hungary; University of South Carolina, UNITED STATES

## Abstract

The problem of soil degradation has accentuated over recent decades. Aspects related to soil erosion and its relation to changes in land use as well as anthropogenic influence constitute a topic of great interest. The current study is focused on a soil erosion assessment in relation to land use activities in the Pănăzii Lake catchment area. Fallout radionuclides were used to provide information on soil erosion as well as redistribution rates and patterns. Variations in the sedimentation rate of the lake were also investigated as these reflect periods in which massive erosion events occurred in the lake catchment area. The novelty of this study is the construction of a timescale with regard to the soil erosion events to better understand the relationship between soil erosion and land use activities. In this study, 10 soil profiles and one sediment core from the lake were taken. Soil parameters were determined for each sample. The activities of ^210^Pb, ^137^Cs and ^226^Ra were measured by gamma spectroscopy. For low ^210^Pb activities, measurements via ^210^Po using an alpha spectrometer were performed. Soil erosion rates were determined by the ^137^Cs method and the sedimentation rate calculated by the Constant Rate of Supply (CRS) model. A soil erosion rate of 13.5 t·ha^-1^·yr^-1^ was obtained. Three distinct periods could be observed in the evolution of the sedimentation rate. For the first period, between 1880 and 1958, the average deposition rate was 9.2 tons/year, followed by a high deposition period (1960–1991) of 29.6 tons/year and a third period, consisting of the last 30 years, during which the sedimentation rate was 15.7 tons/year. These sedimentation rates fluctuated depending on the main land use activity, which can also be seen in the soil erosion rates that had almost doubled by the time agricultural activities were performed in the area.

## Introduction

The investigation of the historical evolution of erosion processes, caused by different types of land management politics, and the long-term roles they play in the evolution of landscapes as well as socioeconomic dynamics is nowadays one of the main research topics in soil science worldwide [1, 2]. In Europe, in the second half of the nineteenth century, soil degradation became an accentuated and well-known problem that required the development of suitable soil management politics [3]. As a result, in 1988, the “European Society for Soil Conservation” (ESSC) was founded in Leuven (Belgium), which was comprised of scientists from ten different countries [4]. European countries are currently showing significant interest in soil erosion research with many scientific papers concerning this subject being published over recent decades [5]. The research takes into account all aspects related to soil erosion and their relation to soil properties, climatic factors, land use changes and anthropic influence [6, 7].

In Romania, the first study concerning soil erosion which was published in the international scientific literature is a report that describes the geographical characteristics of Romania in 1975 [8]. A real interest with regard to the investigation of soil erosion and management in Romania started in the 90’s. According to the study, a potential factor which can increase soil erosion is privatization because erosion may accelerate as a result of crop farming and overgrazing on steep hills with thin soils [9]. Erosion processes in sub-basins and on slopes as well as the effects of anti-erosion protection were also investigated at the time in the Valea Largă basin [10]. The amount of research and the number of publications concerning soil erosion started to grow in the year 2000.

Soil erosion can be assessed in different ways. Fallout radionuclides can be used to provide information on soil erosion and redistribution rates as well as patterns. The potential for using fallout radionuclides in soil erosion studies has been clearly demonstrated by many studies from different parts of the world [11–18] including in Romania [19, 20].

The most used radionuclides for this purpose are ^137^Cs (T_1/2_ = 30.17 y), ^210^Pb (T_1/2_ = 22.3 y) and ^7^Be (half-life≈53 days). ^137^Cs is an artificial radionuclide that originated from nuclear weapons testing and nuclear power plant accidents. ^210^Pb is a natural radionuclide from the ^238^U decay chain, in the soil surface, it has two sources of provenience. In soil, ^226^Ra disintegrates and produces ^222^Rn, a proportion of the radon produced escapes into the atmosphere and, after the disintegration of its short-lived daughter elements, yields ^210^Pb with a half-life of 22.3 years. This fraction of ^210^Pb falls back onto the soil surface and represents the excess quantity or atmospheric component of the total ^210^Pb. The other proportion of the ^222^Rn gas produced disintegrates inside the soil and produces in situ ^210^Pb, which is considered to be in equilibrium with its parent, ^226^Ra. Compared to the in-situ generated ^210^Pb, the airborne deposition is significantly higher. ^7^Be is a cosmogenic radionuclide and its global average production rates (atoms cm^−2^ s^−1^) are 0.041 (^7^Be) and 0.018 (^10^Be) in the stratosphere as well as 0.027 (^7^Be) and 0.018 (^10^Be) in the troposphere for the solar minimum [21]. By measuring the surface distribution of the mentioned radionuclides, the soil redistribution and erosion rates can be estimated. In this manner, ^137^Cs was used in the present study.

The Universal Soil Loss Equation (USLE) was first developed in the late 1950s by scientists from the U. S. Department of Agriculture (USDA), Agricultural Research Service (ARS), Soil Conservation Service (SCS) and Purdue University [22]. During the 1960s and 1970s, the equation was revised and updated before being renamed as the RUSLE (Revised Universal Soil Loss Equation) by a group of scientists from different U.S. institutions and universities [23].

With advancements in the field of information technology, especially GIS (Geographic Information System), the USLE model was adapted and used in a specific GIS environment [24].

The aim of this study is to examine and place on a timescale the effects of land use (grazing, roads and agriculture) on soil erosion using ^137^Cs and ^210^Pb radionuclides. The catchment area of the natural Pănăzii Lake was chosen to be the site for the construction of a timescale for erosion process events. Variations in the sedimentation rate reflect the periods during which massive erosion took place within the catchment area of the lake. By applying the radionuclide distribution model, eroded sites and areas where soil accumulates can be revealed in the studied area.

In the current study, the USLE model was used in a GIS environment according to the methodology described by several studies [24–27].

## Materials and methods

### Study site

**Fig 1:**
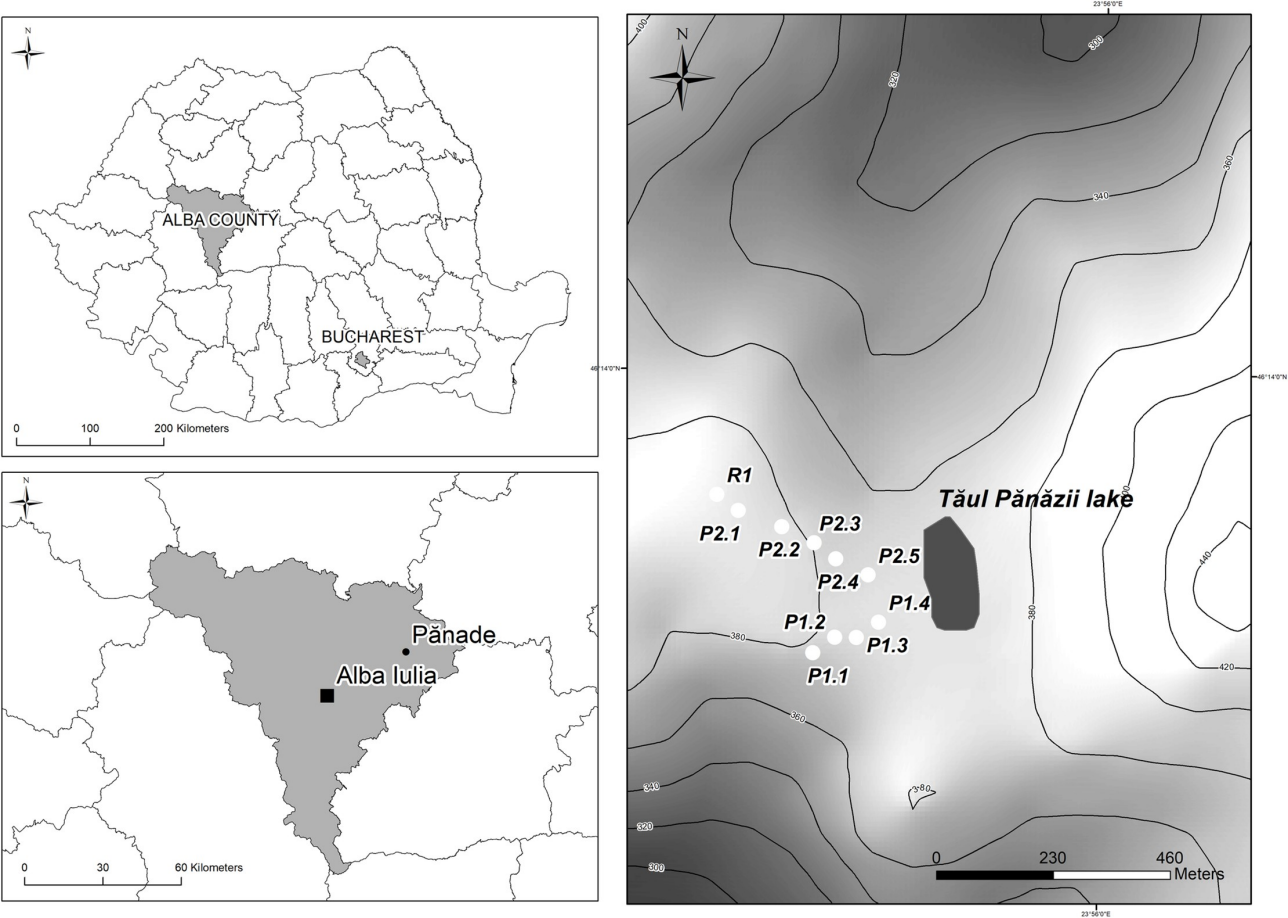
Location of the study site.

Tăul Pănăzii is situated in the western part of the Târnavelor Tableland, a subunit of the Transylvanian Depression (Fig 1). The area is dominated by pastures and arable land, the latter has gradually been abandoned over recent years, thus is converting back into pasture. As a result of land abandonment, the area covered by scrub is also increasing. There are no other major types of land use in the vicinity of the lake. Situated at an elevation of 360 m, the lake covers an area of approximately 1 ha. Drainage occurs to the north towards Pănăzii Creek. Being situated in an agricultural area, this water is consumed by livestock, e.g. sheep and cattle. The catchment area is small and the lake has no direct influx of water from streams. The catchment area of the lake, which was used for agricultural and grazing activities, is 1.2 km^2^ and its surface area is 0.016 km^2^. The position and environmental characteristics in the studied area are suitable for the modeling of anthropogenic land-use erosion processes.

**Fig 2:**
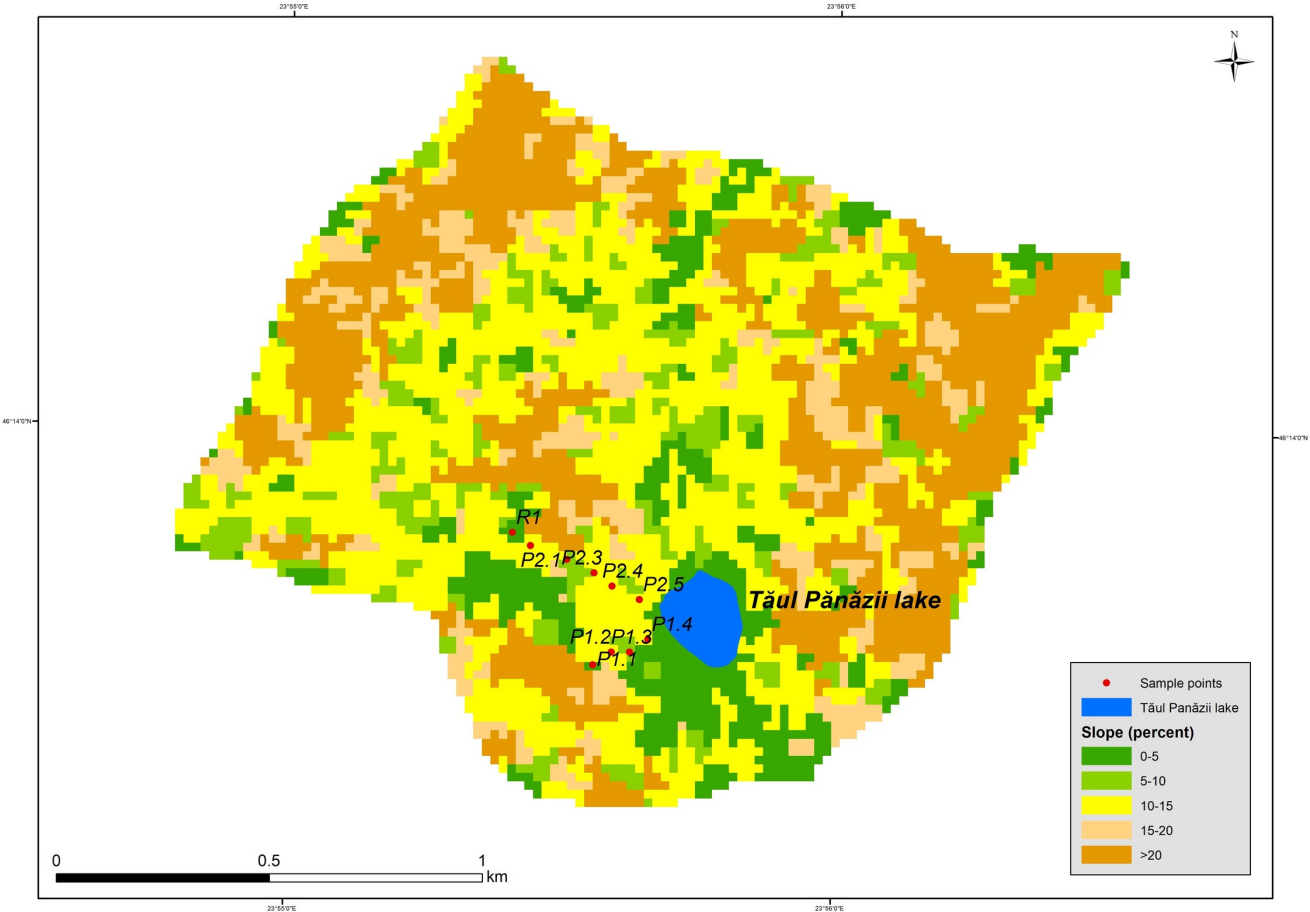
Topological factors of the investigated site.

To investigate the topological effects on soil erosion rates, a map was constructed to represent the gradient and length of the slope with regard to the investigated zone (Fig 2):

### Soil and sediment sampling

No specific permissions were required for the soil and sediment sampling in the catchment area of Pănăzii Lake, as it is not a protected area or private property. No endangered or protected species were involved in the present study.

From the catchment area of Pănăzii Lake, 10 soil profiles and one sediment core from the lake were taken. The soil profiles contained nine short columns (50 cm in depth and sliced into segments 5 or 10 cm in length) and one large one. From the reference site, a column 70 cm in depth sliced into segments 5 cm in length was taken. The short columns are considered as bulk samples. For the study to be representative, two soil sampling lines (paths) from the western part of the catchment area were chosen. The aforementioned sampling line started from two points of the highest latitude on the western part of the catchment area and finished close to the lake at the lowest latitude. From the P1 sampling line, 4 separate columns were taken of between 30 and 50 cm in length. The distance between the sampling points in the P1 sampling line was around 50 cm. The P2 sampling line was drawn in a similar way from 5 different points and the distance between the points was approximately 80 cm (Fig 2).

The reference point was chosen to be a flat area at the highest altitude, where soil erosion is as low as possible. All soil columns were taken using a stainless steel tube with an internal diameter of 5 cm.

One sediment core was extracted from Pănăzii Lake using a gravity corer with an internal diameter of 6 cm and a length of 75 cm. The sediment column was sliced into segments 1 and 2 cm in length.

### Laboratory analyses

#### Soil properties

For measuring the soil pH, a calibrated pH meter was used and the measurements carried out in a 1:2.5 (w/v) soil/distilled water mixture [28]. To categorize the soil according to its organic matter content, Loss On Ignition (LOI) measurements were carried out on each soil column. The soil slices were dried in a drying oven at 105°C to eliminate the remaining water content before being incinerated at 350°C to eliminate the soil organic matter (SOM). After this procedure, only the non-organic matter (non-OM) and carbonates remained in the samples. Once these steps had been completed, the samples were weighed and the parameters calculated. The organic matter content is given by the difference between the weights of the non-organic matter and the dried soil sample [29–31]. Soil particle size analyses were made using a hydrometer analysis method [32, 33]. The particle size fractions obtained were <4 μm (clay), 4–63 μm (silt) and > 63 μm (sand).

#### Radionuclide measurements

The soil and sediment samples were oven dried at 105 ˚C to a constant weight before being ground and sieved through a 2-mm mesh sieve. Each sample was packed in a plastic sample tube 50 mm in length and 10 mm in diameter before being sealed with aluminum tape to prevent the ^222^Rn from escaping from the sample matrix. After being stored for a month (to reach an equilibrium between ^226^Ra and ^222^Rn together with its short half-life radionuclides), the measurements were carried out by using a Well-type High-Purity Germanium (HPGe) gamma spectrometric system (ORTEC GWL-120-15 detector with a resolution of 2.08 keV for 1.33 MeV ^60^Co and 1.1 keV for 122 keV ^57^Co gamma lines). The measurement time in all cases was in excess of 48 hours which was necessary in order to obtain good counting statistics and reach the required maximum ±10% uncertainty in two-sigma confidence intervals. The 46 keV and 661 keV gamma energies were used to determine ^210^Pb and ^137^Cs, while ^226^Ra was determined by using the 294 keV and 351 keV gamma energies of ^214^Pb. To calculate the activity, the relative method was used by keeping the same geometry and matrix between the sample and the certified reference materials (IAEA-312, 327 and 385). The detection limits for ^210^Pb, ^137^Cs and ^226^Ra were 7, 0.3 and 0.5 Bq/kg, respectively.

In some cases, the low activity of ^210^Pb can make precise measurements difficult. In the lake sediment, it was necessary to use alpha spectrometry for the determination of ^210^Pb because of the small quantity of the sample. The total ^210^Pb was determined via ^210^Po, these two elements reach secular equilibrium after 2 years. To 0.5g of each sample, 0.3 ml (100 mBq/ml) ^209^Po was added as a tracer, then each sample was treated with 2 ml HF and 5 ml HNO_3_. After 24 hours, boric acid was added to neutralize HF. By adding 10 ml HNO_3_, the acidic digestion of the samples was continued by using mineral acids (HNO_3_, HCl and H_2_O_2_). The volume of the resultant solution was increased to 100 ml and the pH adjusted by between 0.1 and 0.3. The ^210^Po source was prepared by spontaneous deposition on high nickel content stainless steel discs [34]. The ^210^Po sources were then measured using an ORTEC SOLOIST alpha spectrometer system with Ultra ENS-U900 detectors as well as an active surface area of 900 mm^2^ and a resolution greater than 29 keV. The minimum detectable activity for ^210^Po is 750 mBq/kg.

#### Calculation of soil redistribution and sediment accumulation rates

Soil redistribution rates can be calculated using two models, namely the Point Distribution Model (PDM), which can be used for eroding points in uncultivated soil, and the Mass Balance Model (MBM) for cultivated soils. These models are based on the ratio of the measured ^137^Cs inventory at a specific eroding point to the value of the reference inventory, thus quantifying the relationship between ^137^Cs loss and soil erosion rates. For this study, the PDM was used as the study site is composed of uncultivated soil [35].

The Point Distribution Model (PDM) can be applied using the following function:

$$A^{`}(x)=A_{ref}\left(1-e^{-\frac{x}{h_{0}}}\right)$$ (1)

where *A`*denotes the amount of ^137^Cs above depth *x* (Bq m^-2^),

*A*_*ref*_ stands for the reference inventory for ^137^Cs (Bq m^-2^),

*x* represents the depth from the soil surface (kg m^-2^) and

*h*_*0*_ refers to the coefficient describing the shape of the profile.

Based on the consideration that the depth distribution of ^137^Cs is independent of time and the maximum ^137^Cs fallout occurred in 1963, the erosion rate at a point can be calculated as:

$$Y=\frac{10}{(t-1963)P}ln\left(1-\frac{x}{100}\right)h_{0}$$ (2)

where *Y* denotes the annual average soil loss (t ha^-1^ yr^-1^),

*t* stands for the year of sample collection (yr),

*x* represents the total ^137^Cs inventory lost with regard to the ^137^Cs local reference value (as a percentage),

*A* refers to the measured ^137^Cs inventory at the sampling point (Bq m^-1^) and

*P* is the particle size correction factor.

In order to calculate soil erosion rates in uncultivated soil, h_0_ can be estimated from the vertical distribution of ^137^Cs in the soil profiles at different sampling points using the following equation:

$$A(x)=A(0)e^{\frac{-x}{h_{0}}}$$ (3)

where *x* denotes the mass depth from the soil surface (kg m^-2^),

*A(x)* stands for the ^137^Cs concentration at depth (Bq kg^-1^) and

*A(0)* represents the ^137^Cs concentration in the surface soil (Bq kg^-1^).

Using the soil corer area and the depth of the analyzed core, bulk density can be calculated, which is used to convert Bq/kg in kBq/m2. For the inventory, the numerical integration of the values was applied. Various ^210^Pb dating models based on several assumptions related to the initial activities of ^210^Pb, ^210^Pb flux or accumulation rate [36] exist, which makes it possible to reconstruct variations in the sedimentation rate over time. The model used in this study is the Constant Rate of Supply (CRS) model (also known as the Constant Flux (CF) model). The fundamental hypothesis of the CRS model is based on an efficient transfer from the water column to the sediments and a constant ^210^Pb_ex_ flux at the sediment surface in the absence of diffusion over time. The chronologies are generated by comparing the overall ^210^Pb inventory of the core to partial inventories below depth *i* by integrating the supported ^210^Pb concentration, bulk density and thickness of each sediment layer as well as obtaining the mass sedimentation of each layer in addition to the ages at the limit of each sediment layer [37].

### Using USLE software to estimate soil redistribution

In order to determine the erosion rate, the USLE model was implemented in a GIS environment. This is a straightforward process but it must be taken into consideration that the model consists of several parameters, some of which are fixed.

The USLE is as follows [22, 38]:

A=R·K·L·S·C·P (4)

where

*A* denotes the mean annual rate of soil loss (t·ha^−1^·year^−1^),

*R* stands for the rainfall erosivity factor,

*K* represents the soil erodibility factor,

*Ls* refers to the slope length factor,

*C* is the cover management factor and

*P* denotes the support practice factor.

The equation and all other data processing were done using the ArcGIS 10.3.1 (Esri software) and a Raster Calculator tool.

The rainfall erosivity factor (R) was fixed considering that the study area was small. For the purpose of this study, the standard value proposed in established studies in Romania [39] of 0.12 was used.

The K factor is an expression of the erodibility of the soil or surface material at a particular site under standard conditions [40]. The values considered for this parameter are dependent on the soil type as well as texture and were introduced according to the standard values for the soil type [39].

The slope was used as the basis for the slope length factor (Ls) and was calculated using a 1-meter resolution in DEM (Digital Elevation Model). Determining the slope is straightforward using the slope function implemented in ArcGIS 10.3.1. The slope length factor was determined by using the formula proposed by Mitasova et al. in 1996 [24]. The values of this parameter ranged from 0 to 371 meters. The Cover management factor (C) was obtained by vectorising land-use types based on orthoimagery at 0.5 m resolution. The values for this parameter fell between 0.001 and 2. The support practice factor was not used as no such practices are conducted in the area.

## Results and discussions

### Erosional processes in the catchment area of Pănăzii Lake

The obtained results for the physical and chemical characteristics of the soil are presented in Table 1. According to the obtained values for the soil particle size, it can be assumed that the investigated soil is composed of 24.3% clay, 69.5% silt and 6.3% sand. The average concentration of organic matter of 27.6% reduces the vertical and horizontal mobility of ^137^Cs in the studied layers. The neutral pH value (average of 7.4) of the soil has a positive influence on ^137^Cs migration in the soil due to the presence of water without particle movement.

**Table 1:** Properties of the soil samples in a paddock within the catchment area of Pănăzii Lake.

Sample sites	pH	OM%	non-OM%	Clay (%)	Silt (%)	Sand (%)
P1_1	7.7	30.9	69.1	26.3	68.2	5.5
P1_2	7.5	29.8	70.2	26.2	67.8	6.0
P1_3	7.5	28.4	71.6	24.1	69.9	6.0
P1_4	6.9	27.1	72.9	20.2	73.4	6.5
P2_1	7.8	24.3	75.7	24.3	70.4	5.4
P2_2	7.4	26.6	73.4	25.5	68.3	6.3
P2_3	7.5	29.6	70.4	23.7	69.0	7.3
P2_4	7.0	29.1	70.9	21.9	71.4	6.6
P2_5	6.3	26.0	74.0	25.2	67.7	7.1
R1	7.9	23.9	76.1	25.4	68.6	6.0
*Mean*	*7*.*4*	*27*.*6*	*72*.*4*	*24*.*3*	*69*.*5*	*6*.*3*
*SD*	*0*.*4*	*4*.*0*	*4*.*0*	*3*.*9*	*3*.*6*	*0*.*9*

Where: P1-first sampling line, P2-second sampling line, R-reference point, SD-standard deviation.

In Table 2, the relationships between the investigated parameters are shown. These relationships were determined by calculating the Pearson’s correlation coefficient (PCC). The alpha level (α) was set at 0.05, which is the likelihood of being incorrect when it is assumed that the relationship identified in our sample reflects a relationship in the population. In order to determine if the coefficient calculated for the parameters meets this requirement, the table of critical values for the Pearson’s correlation coefficient was used. These critical values are dependent on the degrees of freedom (DF). The critical value when α = 0.05 and DF = 7 is 0.666, therefore, in the cases where the Pearson’s correlation coefficient exceeds 0.666 or is less than -0.666, a statistically significant relationship exists between the values. The coefficients within this interval are considered to be insignificant. The correlation found between the soil erosion rate and the ^137^Cs inventory is very strong, these two parameters are inversely correlated resulting in the ^137^Cs inventory decreasing if the soil erosion rate increases. This relationship proves the reliability of using the ^137^Cs method for the determination of soil erosion.

**Table 2:** Pearson’s correlation between the investigated parameters of the studied site and the soil erosion rates (t·ha^-1^·yr^-1^).

	pH	OM%	non-OM%	Clay (%)	Silt (%)	Sand (%)	Soil Erosion Rates	Elevation (m)	Slope (%)	^137^Cs Inventory (kBq/m^2^)
pH	1	0.244	-0.244	0.338	-0.121	-0.673	0.130	0.670	0.470	-0.122
OM%		1	-1	0.167	-0.213	0.045	0.324	-0.087	0.440	-0.160
non-OM%			1	-0.167	0.213	-0.045	-0.324	0.087	-0.440	0.160
Clay (%)				1	-0.943	-0.335	-0.043	0.553	0.415	-0.014
Silt (%)					1	0.003	0.213	-0.440	-0.181	-0.207
Sand (%)						1	-0.472	-0.399	**-0.760**	0.624
Soil Erosion Rates							1	-0.118	**0.703**	-0.929
Elevation (m)								1	0.059	0.179
Slope (%)									1	**-0.760**
^137^Cs Inventory (kBq/m^2^)										1

Another strong correlation is between the slope, soil erosion rates and the ^137^Cs inventory. An increase in the value of the slope results in an increase in the soil erosion rates as well as a decrease in the ^137^Cs inventory. These relationships were also validated by other studies found in the literature [41, 42]. Considering the first relationship explained above, any correlation with soil erosion rates has a direct influence and is inversely correlated to the ^137^Cs inventory.

The relationship between the sand content and slope can be explained by the fact that soils richer in sand are not cohesive so are easily detached and transported by runoff. Therefore, the sand content is inversely correlated and decreases as the value of the slope increases [43].

**Fig 3:**
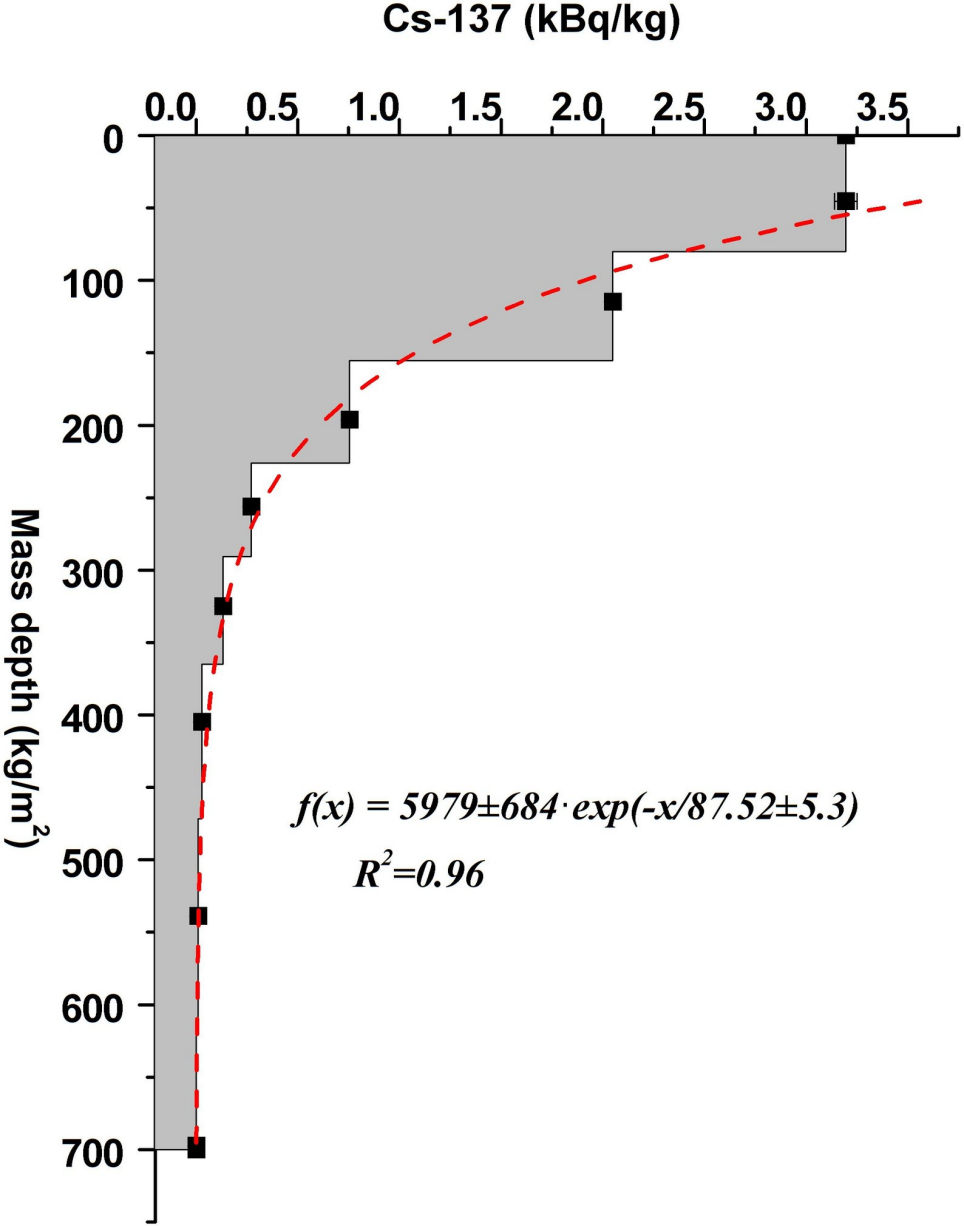
Vertical distribution of ^137^Cs at the reference site and the associated values of h_0_.

**Fig 4:**
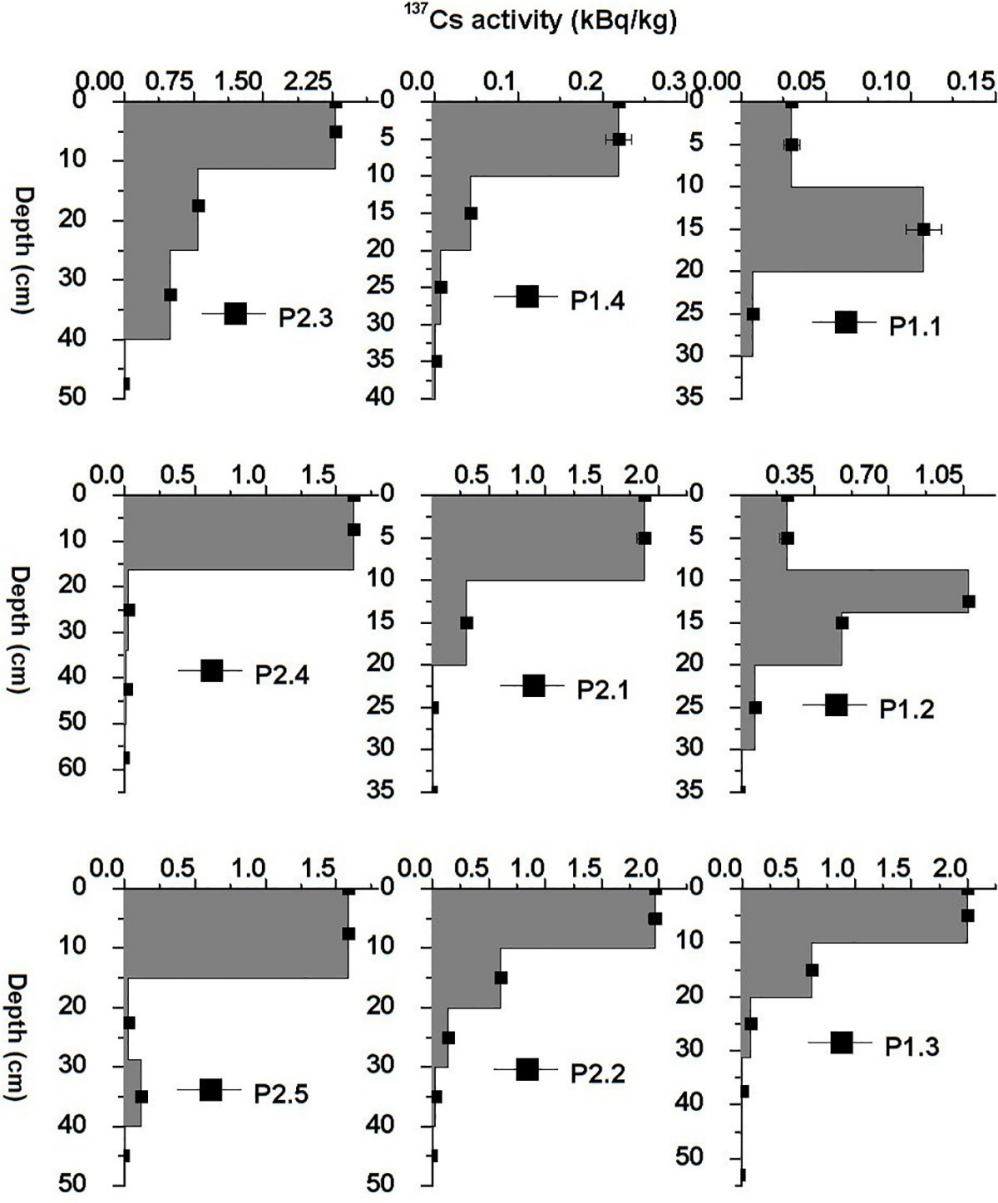
Vertical distribution of ^137^Cs in the soil samples from the investigated points.

**Fig 5:**
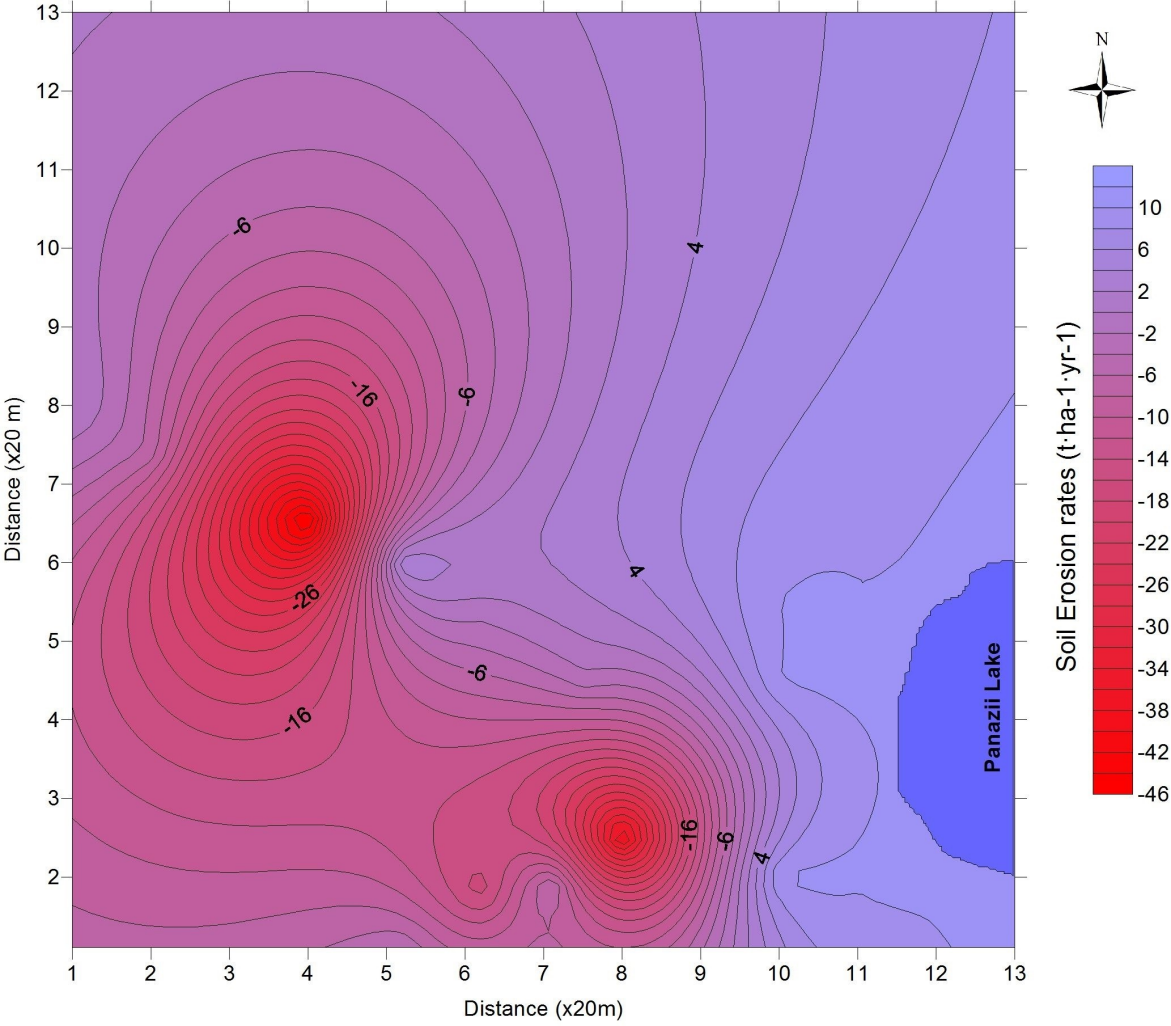
Soil erosion rate for the investigated site.

**Fig 6:**
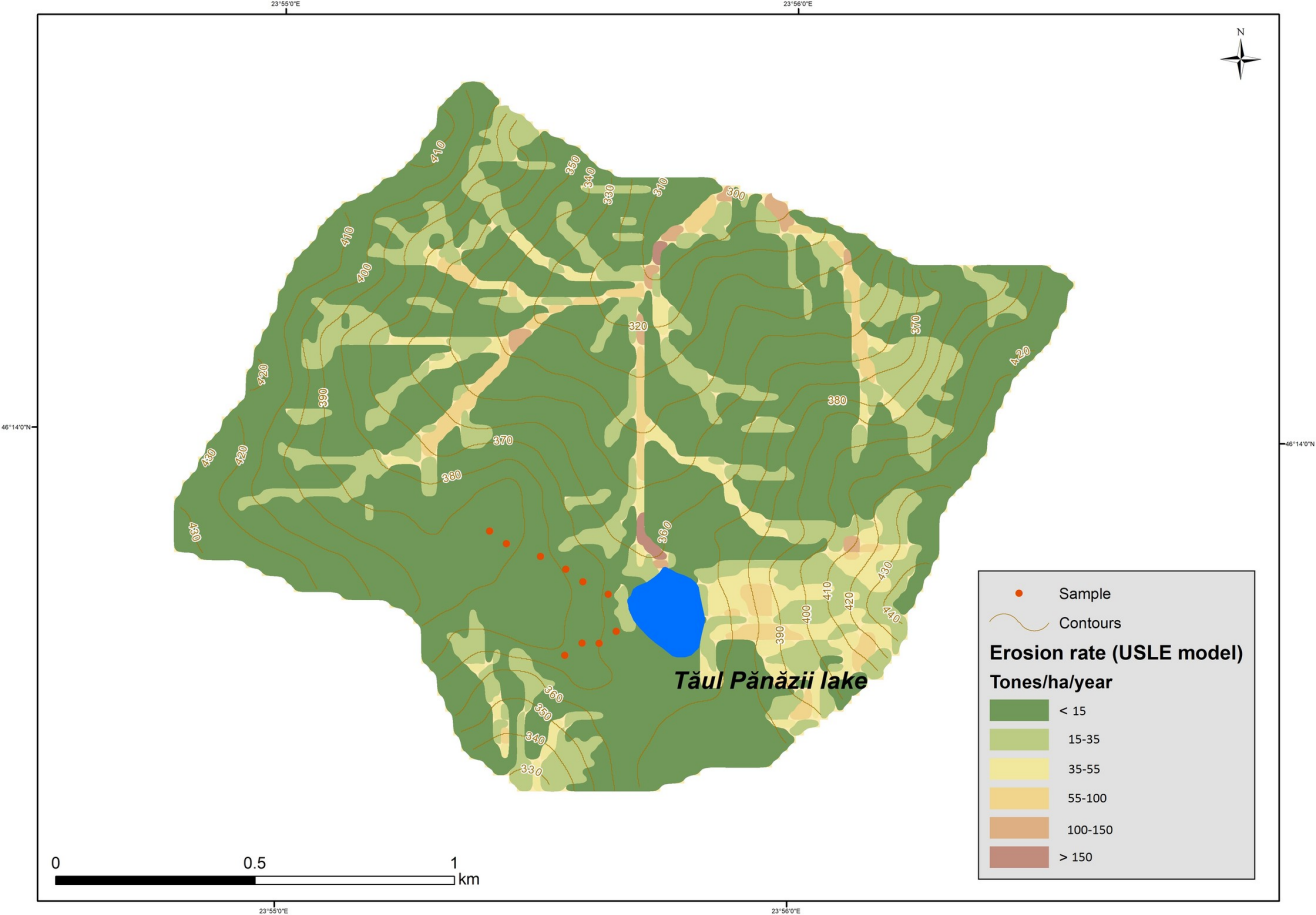
USLE-generated soil erosion rates.

Regarding the investigation of the soil erosion rate, the first step is to select a suitable sampling point from which the core sample will be taken for the determination of the ^137^Cs reference inventory. This is a crucial factor for estimating soil erosion using the ^137^Cs method, the sampling point must be on a flat area and preferably grassland with a slope of zero where no erosion events have taken place since 1954 (the first appearance of ^137^Cs in the environment). For reference sampling point R1, the closest and highest hill was chosen where the erosional processes are minimal according to the topology. The ^137^Cs profile from the core sample proves the suitability of the sampling point. The ^137^Cs activity decreases exponentially, its maximum value is located in the upper part of the core sample. This fact indicates the absence of massive depositions of soil. Furthermore, the obtained value of the total ^137^Cs inventory is similar to the measured values from the region of Transylvania in Romania [44], which denotes the absence of material loss from the reference point. The exponential decrease in the ^137^Cs concentration with depth in column R1 may be due to the downward migration of this radionuclide into the soil [45]. By using the vertical distribution of ^137^Cs activity, which approximated to a first degree exponential function, the parameter h_0_ was determined and its values are presented in Fig 3:

To obtain the values of soil erosion at the investigated points as well as understand the distribution pattern of ^137^Cs, the samples were collected as columns with multiple subsamples and not in bulk form. The distribution patterns of ^137^Cs from each analyzed point are shown in Fig 4. From the vertical distribution of ^137^Cs, it can be observed that only one point, namely P2.3, shows accumulation of eroded soil in which the activity of ^137^Cs is at its maximum. At the other sampling points, the transport of soil containing ^137^Cs is present in the form of erosion. At points P1.1 and P1.2, the erosional processes strongly influence the first 10 cm of the soil. In the remaining core samples, the entire sediment column is affected, the depth distribution is maintained but the value of the total ^137^Cs inventory decreases in comparison to the reference value. The soil erosion rates are shown in Table 3, the mean soil erosion rate for the investigated area is 13.5 t·ha^-1^·yr^-1^. The distribution factor of the soil erosion rate for the analyzed area from the eastern part of the Pănăzii Lake hydrographic basin is shown in Fig 5. It can clearly be observed that the sampling points of maximum erosion are strongly correlated with the slope of the area. According to the simulations performed using the USLE program (Fig 6), the eastern part of the hydrographic basin exhibits a much higher soil erosion rate due to its slope, which is approximately twice as high as that of the western part, and human activities, mainly agriculture, performed in the area. Over the past 10 years, the land-use activity in the area has been grazing in the eastern part of the basin but in the western part, a walnut orchard was planted which will gradually reduce the erosional processes in that area.

**Table 3:** Summary of soil erosion rates within the Pănăzii lake catchment.

Code	Coordinates	Elevation (m)	Slope (%)	^137^Cs Inventory* (kBq/m^2^)	Erosion (%)	Soil Erosion rates (t·ha^-1^·yr^-1^)
*R1 (reference site)*	46^o^13’51”, 23^o^55’19”	410	0	378±22	-	-
*P1*.*1*	46^o^13’41”, 23^o^55’28”	393	19	18±2	35.2	-47.5
*P1*.*2*	46^o^13’42”, 23^o^55’30”	381	15	127±8	66.4	-17.1
*P1*.*3*	46^o^13’42”, 23^o^55’32”	371	13	263±18	30.54	-5.7
*P1*.*4*	46^o^13’43”, 23^o^55’34”	365	9	31±3	91.8	-39.1
*P2*.*1*	46^o^13’50”, 23^o^55’21”	400	10	244±17	35.5	-6.8
*P2*.*2*	46^o^13’49”, 23^o^55’25”	393	5	290±21	23.4	-4.2
*P2*.*3*	46^o^13’48”, 23^o^55’28”	390	4	483±36	-27.8	3.8
*P2*.*4*	46^o^13’47”, 23^o^55’30”	377	6	324±28	14.4	-2.4
*P2*.*5*	46^o^13’46”, 23^o^55’33”	374	6	316±27	16.4	-2.8
*Maximum*				483±36	91.8	3.8
*Minimum*				18±2	-27.8	-47.5
*Mean*				233±18	31.8	-13.5
*Standard deviation*				150	33.5	17.9

* represents the values measured in the year 2019.

Considering that the mean soil erosion rate obtained through the ^137^Cs method is representative of the whole hydrographic basin, the mass of the migrating soil is 1620 t/yr. From this value, 84 t/yr corresponds exclusively to the ^137^Cs investigated area, 540 t/yr to the western area of the basin and 1164 t/yr to the eastern part. For an average slope of 9.6%, the ratio between soil erosion rates from agricultural and pasture land was found to be 2:1. These results can be extrapolated to other areas for the determination of the ratio of soil erosion rates resulting from different land-use activities using the values of the slope. These values validate USLE-generated simulations.

### Lake sedimentation rates

#### ^226^Ra, ^210^Pb and ^137^Cs concentration in the sediment column

**Fig 7:**
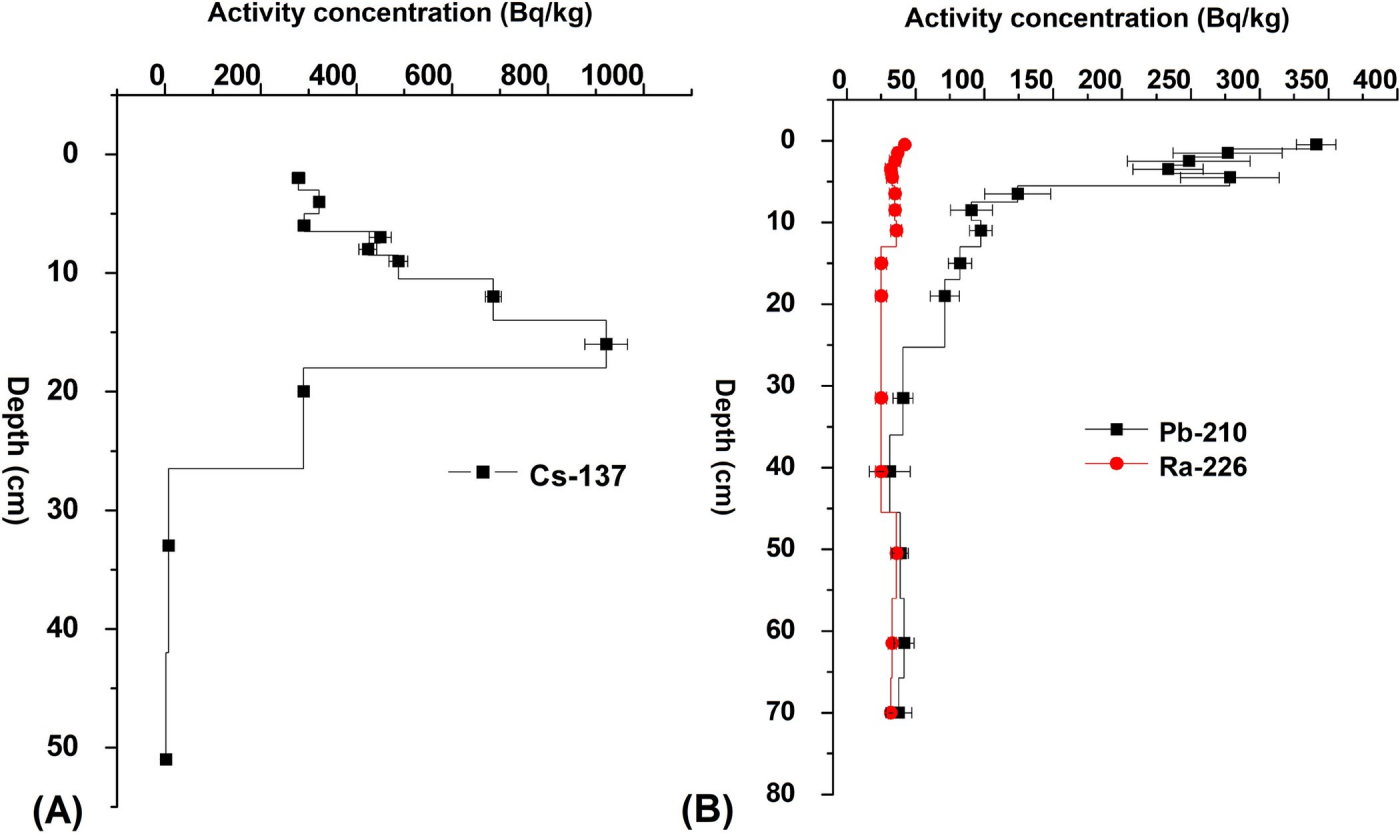
^226^Ra, total ^210^Pb and ^137^Cs concentrations in Pănăzii Lake. (A) represents the ^137^Cs distribution in the lake sediment, (B) represents the total ^210^Pb and ^226^Ra distributions in the lake sediment.

Pănăzii Lake or Pănade Lake is isolated and exhibited an exponentially decreasing tendency with regard to the concentration of ^210^Pb from 346±23 to 45±4 Bq/kg in the first 40 cm. The ^226^Ra concentration was between 25±3 and 49±4 Bq/kg and its dating horizon was around 40 cm in depth (Fig 7B). According to the distribution of ^137^Cs in sediment from Pănăzii Lake, the maximum concentration of 920±30 Bq/kg is located in the layer situated at a depth of 17 cm (Fig 7A):

#### The age of the sediment core and the sedimentation rate

**Fig 8:**
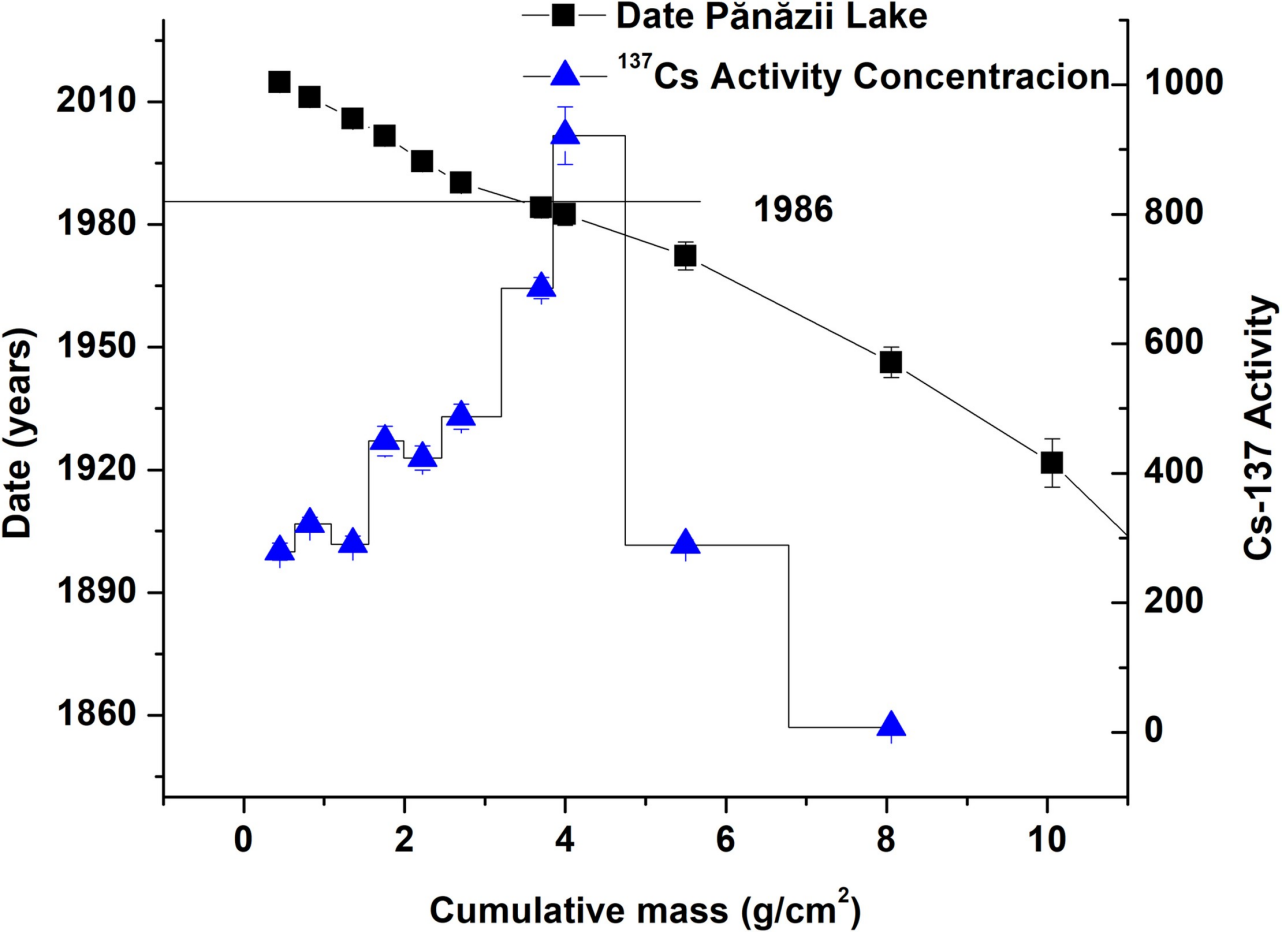
The age-depth model with ^137^Cs activity concentration.

**Fig 9:**
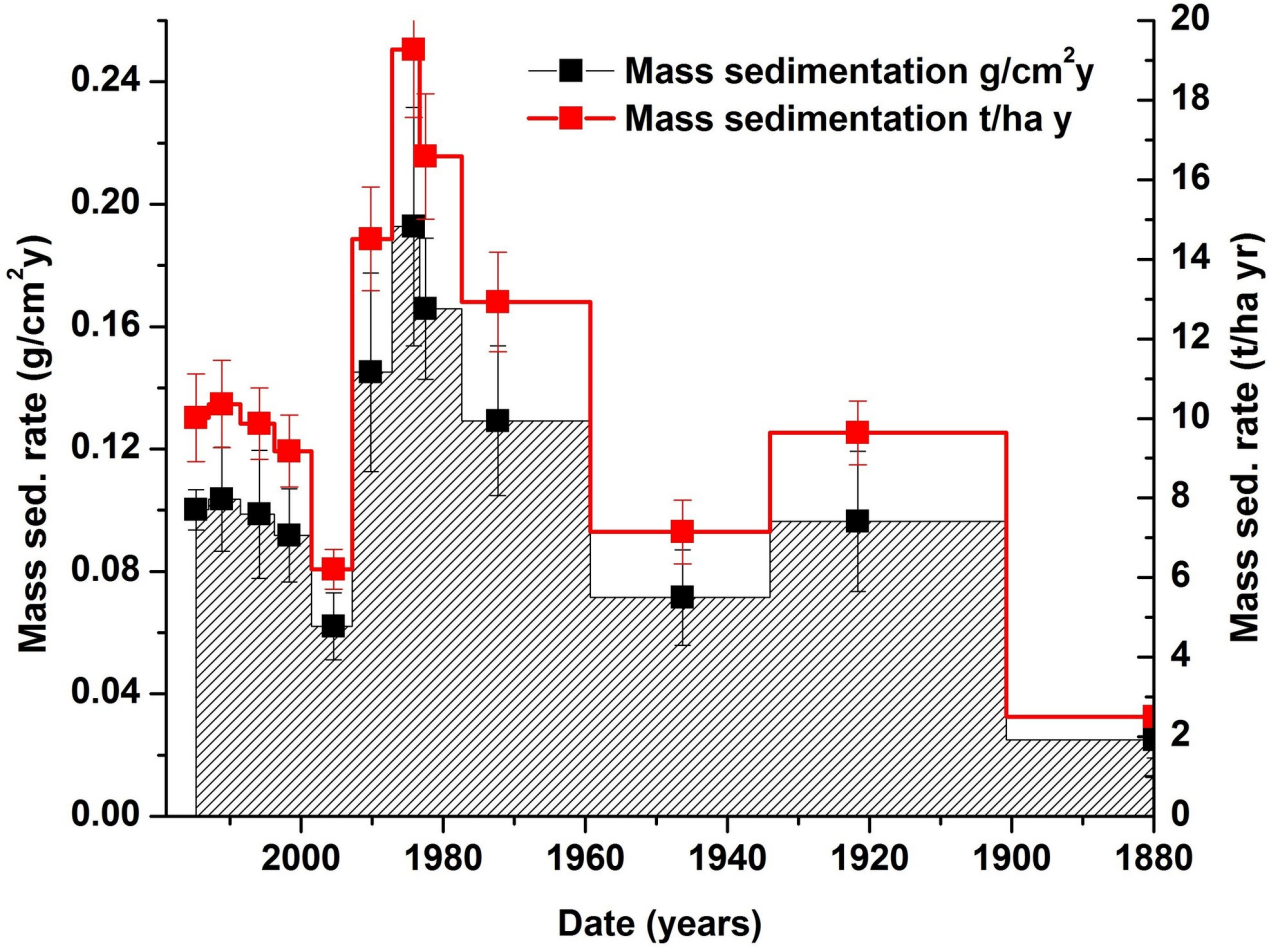
Mass sedimentation rate in Pănăzii Lake.

The age-depth model for Pănăzii Lake is strengthened by ^137^Cs data, which indicates the year of the Chernobyl nuclear disaster, namely 1986. The period covered is from 1880 to 2019 (Fig 8) which consists of three periods of land-management changes and the afferent sedimentation rate calculated by applying the CRS model as can be seen in Fig 9. From the sedimentation rates, the three aforementioned periods are clearly visible. Sedimentation in the lake during the investigated period starts with a relatively low sedimentation rate of 0.064±0.002 g/cm^2^/y on average followed by a period with a high sedimentation rate of 0.158±0.018 g/cm^2^/y on average. The final period, which covers the last 30 years, is described by a moderate sedimentation rate of 0.091±0.008 g/cm^2^/y. Since 1880, the total mass of sediment deposited is 1742 tons which was calculated by determining the total amount of material deposited from calculations using the sedimentation rates and their intervals. Between 1880 and 1972, 48% of the sediment reached the bottom of the lake (844 tons) with a mean deposition rate of 9.2 tons/year followed by a period of high deposition (1972–1991) where 562 tons of sediment was deposited at a rate of 29.6 tons/year. Over the last 30 years, the amount of sediment deposited was 457 tons with a deposition rate of 15.7 tons/year. The sedimentation rate of 10 t·ha^-1^·yr^-1^ for the final period was calculated by the CRS model, which covers 4 years of sediment deposition (Fig 9). Comparing the sedimentation rate of 10 t·ha^-1^·yr^-1^ with the average soil erosion rate of 13.5 t·ha^-1^·yr^-1^, it can clearly be seen that the values are similar and due to the surrounding vegetation of the lake, the amount of sediment that reaches the lake is less than the mass of eroded soil. As a mass balance between the total amount of eroded soil (dislocated soil, of 1620 tons/year from the entire catchment area) and the amount of deposited sediment (16 tons/year), it can be said that just a hundredth of the soil settles on the bottom of the lake. The rest of the soil is redeposited or transported by Pănăzii Creek.

The three distinct periods of sedimentation in the lake are connected to the land use politics of the hydrological basin. On this site, up until 1960, a moderate amount of grazing activity was present, but after 1960, the political regime changed and, as a consequence, the land use management changed too. A massive amount of agricultural activity started to develop on the site and the associated activities caused massive erosion which induced an elevated sedimentation rate in the lake. These significant land use politics dominated the whole of the Romanian lowlands. A new political change occurred in 1989, followed by the return of grazing activity in the area, which produced almost the same sedimentation rate in the lake as was seen from 1880 to 1960. Between 1990 and 1995, the sedimentation rate in the lake rapidly decreased to a value close to the one recorded in 1900. The gap in values of the sedimentation rate is attributed to a short period in which the study site was left as fallow land during which vegetation prevented erosion. By considering that the sedimentation rate rose during the period dominated by agriculture and comparing its value with the actual one, it can be estimated that its value doubled, reaching a soil erosion rate of 26 t·ha^-1^·yr^-1^. Similar tendencies in terms of sedimentation rates can be found in many other lakes in Romania, whose hydrographic basins suffered from massive anthropogenic impacts [46].

## Conclusions

Soil degradation has become an accentuated and well-known problem over recent decades and required the development of suitable soil management policies. Aspects related to soil erosion and its connection with soil properties, climatic factors, land use change and anthropic influence constitute a topic of international interest for researchers.

The current study focuses on the assessment of soil erosion in relation to land use activities in the catchment area of Pănăzii Lake. For this purpose, fallout radionuclides were used to provide information on soil erosion and redistribution rates as well as patterns. Variations in the sedimentation rate in the lake were also investigated as these reflect periods in which massive erosion took place in the catchment area of the lake.

The novelty of this study is the construction of a timescale for the events of the erosional process in order to correlate them with the form of land use which corresponds to different periods of time. By comparing the actual soil erosion rate (13 t·ha^-1^·yr^-1^) with the one corresponding to the period from 1960–1989 (26 t·ha^-1^·yr^-1^), when agriculture was the main land use activity, it is observed that the erosion rate increases by 92% to the actual erosion rate. These values are proof of a strong correlation between soil erosion rates and land use activities performed in the area.

The USLE simulations indicate that the eastern part of the hydrographic basin exhibits a higher soil erosion rate than the western part as a result of the human activities performed in the area and the average slope of 9.6%, which is twice as high as that of the western part. The ^137^Cs method validates the USLE simulation and indicates that from the total mass of migrating soil (1620 t/yr), only 33% is attributed to the western part of the basin, thus the remaining 77% is caused by the erosional processes from the eastern part. The relationship between the gradient and soil erosion rate is validated by the coefficients resulting from the calculated correlations. From the average slope and the ratio between soil erosion rates on agricultural and pasture land suggested in the present work, these results can be extrapolated to other areas for the determination of this particular ratio.

It can be concluded that the determination of the events of erosional processes as well as the construction of a timescale to represent their relationship to different land use politics adopted in the past are compulsory for a thorough understanding of the aspects related to soil erosion, moreover, for the development of suitable land management policies.
